# Current scenario, challenges and way forward for augmenting tobacco control policies and programs in India: a community-based qualitative study

**DOI:** 10.1080/16549716.2025.2491195

**Published:** 2025-05-15

**Authors:** Muralidhar M Kulkarni, Manpreet Bains, Veena G Kamath, Shalini Bassi, Monika Arora, Kirthinath Ballala, Rohith Bhagawath, Priyanka Bantwal, Ilze Bogdanovica, John Britton

**Affiliations:** a Deptartment of Community Medicine, Kasturba Medical College, Manipal Academy of Higher Education, Manipal, India; b UK Centre for Tobacco and Alcohol Studies, University of Nottingham, Nottingham, UK; c Health Promotion Division, HRIDAY, Delhi, India; d Health Promotion Division, Public Health Foundation of India and HRIDAY, Delhi, India; e Public Health, Faculty of Medicine & Health Sciences, University of Nottingham, Nottingham, UK; f UK Centre for Tobacco and Alcohol Studies, University of Nottingham, UK

**Keywords:** Policy, tobacco control, qualitative study, stakeholders, community based

## Abstract

**Background:**

Tobacco use has resulted in a staggering number of illnesses and premature deaths worldwide. India has the world’s second-highest level of tobacco consumption. The study aimed to explore the reasons of initiation among adolescents and understand community stakeholders’ perceptions about the current tobacco control policies and challenges faced in implementing it for youth along with future recommendations.

**Methods:**

Focus Group Discussions (FGDs) were conducted with adolescents in grades 7th–9th, teachers, parents, and police officers, along with in-depth interviews (IDI) with tobacco vendors. These were digitally audio-recorded and transcribed verbatim. Data was analyzed using inductive thematic analysis.

**Results:**

Twenty-two focus groups were conducted with adolescents, 10 with parents, 10 with teachers (*n* = 83), 5 with police (*n* = 42) and 10 tobacco vendors completed one-to-one interviews. Stakeholders identified gaps in tobacco control policy implementation and recommended stricter enforcement. Solutions such as modifying on-screen health warnings, developing novel ways like live demonstration of patients suffering from tobacco use which creates awareness about tobacco harms, countering tobacco industry marketing strategies, restricting tobacco product sales, lowering affordability, and prominently displaying tobacco-free film rules were recommended.

**Conclusion:**

The study provides a thorough understanding of factors that lead to tobacco initiation and stakeholder’s opinion on youth-related tobacco legislation that provides direction for strengthening existing tobacco control efforts. There is a need for novel ways to educate the child’s microenvironment, specifically in school and family environment. The findings also emphasize the importance of multi-sector involvement and better enforcement of tobacco control laws.

## Paper context

- **Main findings**: Strengthen the existing tobacco control initiatives like strict adherence to tobacco-free film and television guidelines, enhanced media advocacy campaigns and policy emphasizing greater emphasis on child’s micro environment.
- **Added Knowledge**: The current study provides perception of community-level stakeholders and emphasize the need for committed multi-sector involvement for implementing the tobacco control laws and improvising the school-based anti-tobacco education.
- **Global health impact for policy and action**: This article provides evidence to reform the existing tobacco control laws and galvanize the implementation of these laws to further reduce tobacco use and move towards a tobacco-free generation.

## Background

Globally, more than eight million people die from tobacco use each year [1]. Over seven million of those deaths are directly linked to tobacco use, and about 1.2 million deaths are due to second-hand tobacco smoke exposure [1]. Over the years, the tobacco epidemic has led to a staggering burden of illnesses and premature deaths across the globe [2].

Globally, tobacco use invariably begins during adolescence [3,4], with 50% of adult smokers reporting initiation before the age of 18 [5]. In India alone, nearly 1 in 10 adolescents aged 13–15 years have ever smoked cigarettes and almost half of these report tobacco use initiation before 10 years of age [6]. Among those adolescents who experiment with smoking, between one-third and one half become regular smokers [7]. The health risks of tobacco use are highest among those who start early and continue its use for a long period [8].

There is sufficient evidence on the predictors of, both smoking and smokeless tobacco use among youth [9,10]. Sociodemographic, psychological, social, and environmental factors [11], pro-tobacco marketing strategies [12], and exposure to cigarette advertising and promotion via media [13] increase tobacco use among adolescents [14]. Exposure to smoking in public places and tobacco depictions in films, television, and videos are considered to increase the risk of smoking uptake among youth [12,15]. It is also linked to parental smoking, a lack of parental control, peer pressure, poor school grades, and a lack of school connectedness [16].

Preventing tobacco uptake is a public health priority across the world, including India. The government has enacted the Cigarettes and other Tobacco Products Act (COTPA) [17] in 2003 with timely amendments, and a National Tobacco Control Program [18] that started in 2007. These policies cover aspects like school programs, capacity building, monitoring tobacco control laws, and providing support for cessation across states and districts of the country. Guidelines for Tobacco Free Educational Institution (2017) were also developed to scale up the tobacco control efforts at schools, colleges, or any other higher educational university [19].

The way ahead includes effective monitoring and stringent implementation of these tobacco control policies, steep increases in taxes on all tobacco products [20], a reduction of the tobacco cultivation area, and compensation for the employment lost due to tobacco control policies by offering employment opportunities to workers in other labor intensive services. Measures that have worked in the country include hard-hitting anti-tobacco advertisements [21], graphic pack warnings [22], and tobacco-free film and TV rules [23]. Other factors such as bans on tobacco point-of-sale display and tobacco prevention programs in schools are also considered protective [24,25]. While these efforts have been effective, there are challenges in the enforcement of relevant regulations [26].

The Global Adult Tobacco Survey [27,28] in India has shown a decline in tobacco use among adults. Similarly, the Global Youth Tobacco Survey (2019) [6,29] has also reported a decline in tobacco use among adolescents in the country from 14.6% to 8.5%.

Adolescence is an at-risk population. Although these reductions are encouraging, there is no scope for laxity but to carry the momentum forward to devise effective strategies to combat the tobacco problem among this vulnerable group. Hence, the current study was undertaken to understand the reasons for initiation of tobacco use, perceptions and challenges faced by community stakeholders towards the current tobacco control policies for youth, and recommendations to address the tobacco use behavior among them.

## Methods

### Design

This was a qualitative study comprising semi-structured focus group discussions with adolescents in 7^th^ to 9^th^ grades (10–15 years), teachers, parents and police constables and one-to-one in-depth interviews with tobacco vendors.

### Study population, recruitment, and sample size

Adolescents were recruited from 914 schools using purposive and convenience sampling across five educational blocks in Udupi district through head teachers after briefing them about the study. Sampling of schools was done with representation from urban and rural area and those that were public, private, or part funded, to ensure representation of participants across district. Once the school authorities agreed to take part in the qualitative study, 12 students were identified to participate as recommended by the respective class teachers. In line with previous research on gender differences [30] in relation to smoking initiation, gender-specific FGD were conducted with adolescents and parents. Similarly, for parents we requested the head teacher to identify the students whose parents were interested and can take part in the discussion and likewise for teachers. In the case of police constables, the staff in-charge of the station in each block was requested to list the participants who can take part in the discussion. Tobacco vendors were randomly selected from each block across the district. Thus, we had representation from all blocks of the district across all participant groups.

### Data collection procedure

Semi-structured topic guides (Supplementary file 1) were developed for each stakeholder group based on the literature and study aim (by SB, MB) and reviewed by MA, VGK, JB, MMK, and KB. Broad topics were covered with all groups, such as understanding on tobacco use, specifically among young people, knowledge of tobacco-related harms, prevention of uptake, and smoking cessation. Perceptions regarding existing tobacco control laws, tobacco control activities in schools, tobacco control efforts in state/district, the key challenges, opportunities, and recommendations to further augment existing policies and programs were also covered. Data were collected between July 2019 and January 2020 by a team of 13 researchers trained in qualitative research.

All stakeholder groups were provided with a participant information sheet and had the opportunity to ask questions, prior to providing consent. For students, information sheets were shared a week in advance and parental consent was sought. Each FGD/interview was conducted in a separate quiet premises in the presence of two researchers where one led, while the other moderated and took notes. Before starting, written assent from adolescents and consent from parents, teachers, police constables, and vendors was obtained.

Participants were assured about confidentiality and anonymity regarding data management and their right to withdraw from the study before the commencement of FGD or interview. FGDs for adolescents, teachers, and parents were conducted in schools. The FGDs among police constables were conducted in the police station, while the interviews with vendors were conducted at the time convenient to vendors in the shops. FGDs and interviews were digitally audio-recorded and transcribed verbatim by the research team, in the language in which they were conducted (Kannada/English). The interviews in local language (Kannada) were translated into English and also back translated to ensure quality and accuracy. Each transcript was checked to ensure accuracy and that personal identifiers were removed. Data collection was terminated once theoretical saturation was attained as additional data no longer yielded any new insights.

### Data analysis

The data was analyzed thematically, adopting deductive and inductive approaches, led by VK and MK and overseen by MB and SB [31,32]. NVivo 12 was used to manage data and facilitate analysis. Further, the personal identifiers of participants were removed to ensure anonymity. First transcripts from student FGDs were read closely which facilitated data familiarization. Subsequent readings led to the noting of initial codes, where further analysis led to the generation of more substantive themes and sub-themes. MB and SB double-coded data (investigator triangulation) and compared themes and sub-themes with the team working within the study district (MK, VK and RB). This led to refinements and agreement on the final set of themes. Data from other stakeholder groups was coded successively, where themes identified from student groups applied and additional stakeholder specific ones were added; this also facilitated comparison between groups and within. MB and SB double-coded a sample from each group to ensure validity. As it was an exploratory study, no conceptual framework was utilized.

## Results

### Participant characteristics

Twenty-two FGDs were conducted with adolescents (*n* = 181; male = 95, female = 86), 10 with parents (*n* = 78), 10 with teachers (*n* = 83), 5 with police (*n* = 42), and 10 tobacco vendors participated in one-to-one interviews (Supplementary file 2). Each group comprised approximately 8–10 participants. Four themes along with corresponding sub themes were identified as shown in Table 1. Extracts are presented throughout with a code which signifies: T/S/P/PC/V (Teacher, Student, Parents, Police Constables, Vendors), the numeral represents FGD or IDI code, next letter represents the type of school G/P/A (Government, Private, Government aided), and the last character identifies the gender M/F (Male, Female).

**Table 1.** Qualitative themes.

Themes	Sub-Themes
Reasons for initiation of tobacco among adolescents	• Peer pressure • Familial smoking • Curiosity • Role of media • Tobacco industry advertising
Awareness and Perception towards youth related tobacco control policies	• Sale prohibited in 100 yards of educational institution • Minimum age for sale of tobacco products • Display of health warnings in movies • Ban of smoking in public place • Awareness regarding NTCP activities, COTPA and other tobacco control laws
Existing school-based tobacco control initiatives	• Raising awareness on harms of tobacco • Display of tobacco prohibition board
Recommended strategies to address tobacco use among the youth	• Improved implementation • Activities to generate awareness in school setting • Tobacco imagery and mass media

#### Reasons for tobacco initiation among adolescents

Peer pressure and familial tobacco use were referred to most across stakeholder groups as reasons for tobacco initiation among adolescents. Vendors shared: ‘*They do not realize the problems and they continue doing in their own small groups- it’s like learning from one another’ (V5)*. Some students perceived ‘*through teasing and mocking, they can force others to use cigarette’ (S4)*. A teacher also stated, ‘*If the family members use beedi or cigarette, automatically children follow them. They start using out of curiosity*’ (T26GMF). Some students shared that some family members, such as ‘*Grandfather not only asks children to buy cigarettes but also teaches his grandson how to use them*’ (S8GM).

The affordability and availability of tobacco and smoking being perceived as fashionable were also cited as reasons for tobacco initiation and subsequent addiction among adolescents. Adolescents shared, *‘poor people try to imitate rich people because they believe if they eat costly tobacco, they can improve their status.’*

All stakeholder groups highlighted the role of the media in tobacco uptake among adolescents. Participants mentioned that adolescents viewed actors (in TV and film) as role models, and thus they modeled behaviors depicted onscreen, including smoking: ‘*Fifty percent of people initiate tobacco consumption only because of TV and movies, when they see actors and actresses using tobacco’* (S4AF). Students also mentioned that various social media platforms such as Facebook and WhatsApp, along with news portals, were factors influencing tobacco use initiation.

Tobacco industry advertising targeting young people was also referred to along with vendors employing sales tactics targeting adolescents. ‘*To increase the sales, the shopkeeper gets tobacco products when teenagers come to buy chocolates, they tell them that this product is good and to try it*’ (S6PM). Some even shared that *‘Shopkeepers also commit the mistake of hanging the smokeless tobacco packs in front of their shop’* (S11GF) which may lead adolescents to try products out of curiosity.

#### Awareness of and perceptions on youth-related tobacco control policies

Most participants were aware that the sale of tobacco was prohibited within 100 yards of any educational institutional area. However, some parents and teachers mentioned that despite this policy, tobacco was still available, and that enforcement efforts dwindled over time, which was echoed by police officers. *‘Only in the initial days they will see if it is being followed or not. Later they don’t come and check to see if they are selling the products*’ (PC30M); this laxity applied to all other policies as mentioned by most participants.

Minimum age of sale was routinely cited as a youth-related tobacco control policy. However, adherence, particularly among vendors, was refuted by students. ‘*In some shops they sell tobacco products to minors’* (S4AF). On this the vendors shared, *‘If young people come here to buy tobacco, we do not sell tobacco to them. We sell tobacco products but what to do! If we don’t sell, they will purchase it from other shops. So, we need to sell tobacco products in our shop’* (V3).

Health warnings displayed in movies were criticized for being too small and thus were unlikely to divert a child’s attention from the smoking depicted onscreen; they ‘*are not at all useful’* (T39PMF). Some students were aware of these disclaimers, but felt they were ineffective. Most participants perceived *‘adolescents paid more attention to the drinking and smoking behaviours, rather than the warning messages’* (P44CF). However, participants highlighted that in films, *‘they give a message at the bottom of the screen that smoking is injurious to health, they show scenes which includes smoking and drinking simultaneously’* (P24CM).

Participants had contradictory views on the ban on smoking in public places; some felt ‘*after introducing COTPA, there is a reduction in smoking in public places’* (PC30M), but others mentioned that despite a fine of Rs.200, *‘ … people now smoke in the public places even when police are around*’ (T25PM). Other policies were referred less often; for instance, warning messages on tobacco packaging were considered ineffective and awareness of quit line number appeared poor.

Awareness of the National Tobacco Control Program (NTCP) was low among all stakeholder groups; some expressed ignorance stating, ‘what is that!’ (P24) except police constables who had a fair amount of knowledge about the program but shared that ‘*the training is done for the higher ranks in police force; but not for the lower ranks*’ (PC31MF).

#### Existing school-based tobacco control initiatives

Most of the school-based initiatives appeared to focus solely on raising awareness of tobacco-related harms with less emphasis on tobacco control policies. Many students commented on completing some form of quiz or competitions that sought to raise awareness on the harms of tobacco use, or departments such as the police and personnel from the governmental health sector had delivered talks on the topic. ‘*Asha workers (Accredited Social Health Activist in Government sector) organize programs, wherein information is provided to youngsters about problems of tobacco addiction*’ (S7AF).

Many teachers mentioned that their schools displayed tobacco prohibition boards and stated it was a ‘*tobacco free school*’ (T18PM) or that was a ‘*tobacco prohibited area*’ (T18PM). Some respondents also cited that their schools exhibit boards on the prohibition of tobacco use or sale within 100 yards of educational institutions, which seemed to be monitored in some instances, by some schools; ‘*we have very well followed the instruction and made sure no shops sell tobacco products within the 100-meter distance around the school’ (T26GMF)*. The vendors also ascertained that *‘Officials from the station (police station) visited our shop two years ago to ask us to put the board compulsorily’ (V3M).*

### Recommended strategies to address tobacco use among youth

#### Improved implementation

Participants from each group felt that heavy fines must be levied on those who smoke or consume tobacco in public places. Vendors also stated that *‘if police are strict and take immediate action on smokers for smoking in public places, cigarette and tobacco use can be controlled’* (V4M). Other suggestions advocated for strengthening of current policies, ‘*only the license holder must be allowed to sell*’ tobacco (PC30M), which could help in regularization of sales of tobacco products.

Police personnel also suggested setting targets for filing a certain number of violations per month, as a way to improve monitoring of tobacco control laws. Officials from the police department also felt a multi-sectoral approach was warranted. ‘*All departments like the revenue and education department should come together to control tobacco’* in addition to which they also suggested representatives of ‘*block level, district level, village level should come together’* (PC34MF).

#### School setting

Across FGDs, the police suggested that the NTCP must extend its activities encompassing school-based sessions on tobacco harms by creating awareness among families. In line with this, parents also felt that schools need to work better with them, and that there is a *‘need to have open discussion on tobacco impact among children’* (P22PF).

Others stakeholders believed, ‘*Children do not have knowledge of impact of tobacco, so there should be a new syllabus on tobacco topics at school and should not be changed for any reason*’ (P22PF). Similar views were echoed by adolescents, stating that ‘*If children gain knowledge about tobacco, they will educate their family members, neighbors … even tobacco user, if any’ (*S17GF). Timing of delivery was also debated, ‘*It’s better to take as a subject in Secondary School Leaving Certificate (SSLC) (10th grade), because the children may not have an interest to read when they go to PUC (12th grade, Pre-University Course)’* (P28CF). It was also suggested that real-life impacts of smoking could be integrated into school programs; for instance, *‘Every school has a tour, so students can be taken to cancer hospital as a study tour, that way students will observe the sufferings in the hospitals, their lifestyles and will reinforce them to not get into such bad habits in their life’* (T26GMF). Parents felt awareness sessions on tobacco should be reinforced every year and expressed that ‘*if such programs are conducted every year, it will be effective*’ (P27CF) and ‘*if they make us a part of it (parents), I think it will be useful*’ [33].

#### Tobacco imagery and mass media

Among participants, smoking in movies was considered an influential factor driving tobacco use in adolescents. As such, many participants felt that *‘The usage of smoking in stylish way should be stopped’* (P43CM); *‘they will show the scene where hero is smoking. Such scenes will have a strong impact over the youth. Such scenes must be removed first and then the movie must be certified and released’* (PC30M). Many participants believed there should be reforms introduced in film making, with special emphasis on long-term effects of tobacco consumption. Health issues and financial implications for the treatment of tobacco-related diseases be shown. Some teachers felt ‘*As a teacher, we must not just teach them what’s written in the books but also create awareness among them about the current affairs (ill effects of tobacco) and correct them; We must tell them the difference between the reel life (shown in the movies) and real life’ [25]*. Students also shared *‘showing a short movie about the hazards of tobacco during the film intervals’* (S15PM) could be a way to raise awareness. Change in the content and placement of health warnings such as disclaimers and static messages in movies were also discussed by some students and teachers, *“In movies, instead of writing ‘smoking is injurious to health*’, it would be better if written ‘*Do not smoke’ (S8GM), ‘Instead of putting (static message) it at the bottom, they can put it on the top (of screen)’* (S14PM).

A few students also felt anti-tobacco advertising in newspapers and online platforms could also be important. They repeatedly suggested creating awareness on tobacco through other means ‘*like short films, street plays can also be used to generate awareness*’ (S41GF).

## Discussion

This community-level qualitative study sought to understand the crux of tobacco control issues among youth in the country from the perspective of various grass root stakeholders. The stakeholders pointed out gaps in the implementation of tobacco control policies and pressed for stringent enforcement. Participants suggested various ways to address the matter, including modifying on-screen health warnings, novel school-based education programs focusing on hazards of tobacco use and tobacco control policies, restricting the sales of tobacco products, reducing affordability, and prominent display of tobacco-free film and TV rules on depiction of tobacco imagery in films.

The role of family, peer influence, easy availability to tobacco products, aggressive promotion tactics of the tobacco industry, and vulnerability of young children and adolescents were cited as reasons for initiating tobacco use in line with international literature [34,35]. A cross-sectional survey on adolescents in Switzerland deduced peer and parental model behavior, along with their approval or disapproval, as a key factor influencing initiation of smoking behavior among adolescents [33]. Similar study in Maharashtra, India, concluded that stress (49.2%) along with peer pressure (35.1%) as key reasons for tobacco initiation among students [36]. This calls for a need to focus on the micro-environment- and school-based intervention to tackle peer pressure through novel interventions.

In our study the access to and affordability of tobacco products was perceived to be a critical parameter in tobacco control efforts. This is supported by a study by Goodchild et al. which stated that tobacco products are still economical, with major differences in their absolute prices [37], which can be tackled with increase of taxes across all tobacco products [38].

Active marketing by the tobacco industry targeting youth was stated as the reason for tobacco initiation in the current study. The project titled Tiny Targets India highlighted that multinational tobacco companies and vendors advertised tobacco products near schools, which is a direct violation of the Cigarettes and Other Tobacco Products Act (COTPA) that has been expressed by adolescents and other stakeholders also in the present study [39]. This highlights the need to strengthen the implementation of tobacco control laws on tobacco advertising, promotion and sponsorship.

Stakeholders had contrasting opinion on the youth tobacco control policies and its effectiveness. Similar account has been shared by stakeholders across other districts, thus supporting the findings of the study [40]. They also believed that tobacco control policies need to be amended and more effective implementation is needed. Despite legal provisions, many stated that tobacco is still sold near schools and is even sold to minors. Similar findings were found in an observational study conducted in Delhi which assessed compliance with various sections under COTPA [41] wherein non-compliance to Section 4 (ban on smoking in public place), Section 6a (sale to minors) and 6b (sale within 100 yards of educational institutions) was observed. The evidence from the present study and other studies stresses the need for effective implementation of Tobacco Free Educational Institutions guidelines and calls for inter-sectoral involvement for better enforcement of laws.

The majority of the participants believed that the anti-tobacco messages in media were ineffective because messages were inconspicuous. This is in line with our previous research finding that has reported gross non-compliance to tobacco-free film and TV rules in top grossing films in India [9], and calls for stringent implementation of tobacco-free film and TV rules as the presence of compliant anti-tobacco messages helps in attenuating the impact of smoking imagery by preventing smoking uptake [42].

All stakeholders suggested better implementation of the existing policies to reduce tobacco initiation and discontinue tobacco use. This is evident with aspects like irregular visits by policy implementers, lack of training opportunities for implementers at grass root level, low awareness regarding training programs, and lack of timely follow-up being highlighted by many stakeholders. A review paper on IMPACT Framework [43] (Intervention Model to Protect Adolescents and Children from Tobacco) highlighted community- and policy-level interventions like prohibiting tobacco advertisements, school health programs, mass media campaigns, and restricting access to minors, similar to the themes that emerged in the FGDs and IDIs conducted in our study.

The need to provide comprehensive anti-tobacco education is highlighted in research papers, beyond the ill-effects of tobacco and include aspects like addressing gender differences among smokers, peer support, and life skills training [44–46]. In another research on ever smokeless tobacco (SLT) use, it was proven that good awareness about tobacco harms and tobacco control policies prevents tobacco uptake among adolescents and can address this risk factor [10,47]. Similar findings emerged in our study, where modifying school programs was mentioned by teachers, parents, and other stakeholders to empower adolescents.

## Strengths and limitations

The strength of our study lies in the inclusion of the perceptions of community-based stakeholders, comprising vulnerable adolescents and those involved in enforcement of tobacco control laws. The insights from various stakeholders provide a good opportunity to triangulate the views shared across and within groups, and suggest efforts to address the problem, resulting in substantial impact. As with any qualitative study, participants may provide socially desirable response in agreement with other participants, and this may have a negative impact on the outcome of the study.

## Conclusion

The study provides an in-depth understanding of reasons for tobacco use initiation and people’s perception on the youth-related tobacco policies and programs. Key recommendations to strengthen the existing tobacco control efforts included adherence to tobacco-free film and TV rules, emphasis on increasing the price of tobacco products, stronger media advocacy programs, along with policy emphasizing the micro environment of the child that encompasses of family and friends, levying of increased fine for violations of tobacco control laws along with introducing tobacco vendor license to sell tobacco products. The study findings clearly demonstrate that the multi-sectoral collaboration and improved anti-tobacco education in schools are the most important priorities to reduce tobacco uptake and use among youth.

## Supplementary Material

Supplimentary File 2.docx

Supplimentary File 1_.docx

## Data Availability

Data that support the findings of the study will be available upon request from MMK, after seeking approval from the ethics committee.
