# Supplementary material for: Current scenario, challenges and way forward for augmenting tobacco control policies and programs in India: a community-based qualitative study
Source: Glob Health Action. 2025 May 15;18(1):2491195. doi: 10.1080/16549716.2025.2491195 (PMC12082722; doi:10.1080/16549716.2025.2491195)
Supplement: Supplimentary File 2.docx [file ZGHA_A_2491195_SM8623.docx]

| **Sl. No** | **FGD No.** | **Location** | **Type of Area** | **Educational Block Name** | **Type of FGD** | **Type of School** | **Grade** | **Age** | **Gender** | **Number**  **of Participants** | **Duration of FGD (In Hrs)** |
| --- | --- | --- | --- | --- | --- | --- | --- | --- | --- | --- | --- |
| 1 | S1PF | Indrali | Urban | Udupi | Adolescents | Private | 9 | 14-15 | Female | 10 | 00:46:14 |
| 2 | S2GM | Shettibettu | Rural | Brahmavara | Adolescents | Government | 9 | 14-15 | Male | 8 | 00:46:39 |
| 3 | S3GF | Kirimanjeshwara | Rural | Byndoor | Adolescents | Government | 9 | 14-15 | Female | 10 | 00:39:59 |
| 4 | S4AF | Kundapura | Urban | Kundapura | Adolescents | Aided | 9 | 14-15 | Female | 9 | 01:19:00 |
| 5 | S5GF | Pervaje | Rural | Karkala | Adolescents | Government | 9 | 14-15 | Female | 9 | 01:47:36 |
| 6 | S6PM | Alevoor | Rural | Udupi | Adolescents | Private | 9 | 14-15 | Male | 8 | 01:50:46 |
| 7 | S7AF | Saibrakatte | Rural | Brahmavara | Adolescents | Aided | 9 | 14-15 | Female | 10 | 01:55:49 |
| 8 | S8GM | Heskuthur | Urban | Kundapura | Adolescents | Government | 9 | 14-15 | Male | 9 | 02:02:45 |
| 9 | S9GF | Belman | Urban | Karkala | Adolescents | Government | 9 | 14-15 | Female | 7 | 01:36:34 |
| 10 | S10AM | Mavinkatte | Rural | Byndoor | Adolescents | Aided | 9 | 14-15 | Male | 8 | 01:30:23 |
| 11 | S11GF | Hanumantha Nagara | Rural | Brahmavara | Adolescents | Government | 9 | 14-15 | Female | 7 | 02:17:58 |
| 12 | S12PM | Kodladi | Rural | Byndoor | Adolescents | Private | 9 | 14-15 | Male | 8 | 02:06:35 |
| 13 | S13PM | Kundapura | Urban | Kundapura | Adolescents | Private | 9 | 14-15 | Male | 8 | 01:58:38 |
| 14 | S14PM | Karkala | Urban | Karkala | Adolescents | Private | 9 | 14-15 | Male | 7 | 01:17:40 |
| 15 | S15PM | Parkala | Urban | Brahmavara | Adolescents | Private | 9 | 14-15 | Male | 7 | 00:54:56 |
| 16 | S16GM | Muniyalu | Rural | Karkala | Adolescents | Government | 9 | 14-15 | Male | 8 | 01:36:08 |
| 17 | S17GF | Uppinakudru | Rural | Byndoor | Adolescents | Government | 9 | 14-15 | Female | 8 | 01:22:57 |
| 18 | S35PM | Malpe | Urban | Udupi | Adolescents | Private | 8 | 13-14 | Male | 8 | 01:12:21 |
| 19 | S36GM | Kalmady | Urban | Udupi | Adolescents | Government | 9 | 14-15 | Male | 8 | 01:12:11 |
| 20 | S37AF | Adi Udupi | Urban | Udupi | Adolescents | Aided | 9 | 14-15 | Female | 8 | 01:11:28 |
| 21 | S38GF | Tekkatte | Urban | Kundapura | Adolescents | Government | 9 | 14-15 | Female | 8 | 01:06:31 |
| 22 | S41GF | Tallur | Rural | Byndoor | Adolescents | Government | 8 | 14-15 | Female | 8 | 01:10:52 |
| 23 | P19AF | Basroor | Rural | Kundapura | **Parents** | Aided | - | 40-45 | Female | 6 | 01:22:10 |
| 24 | P21CF | Padukere Malpe | Rural | Udupi | Parents | - | - | 35-45 | Female | 9 | 01:16:22 |
| 25 | P22PF | Belman Nitte | Urban | Karkala | Parents | Private | - | 40-45 | Female | 8 | 01:43:20 |
| 26 | P24CM | Alevoor | Urban | Udupi | Parents | - | - | 40-50 | Male | 9 | 01:36:50 |
| 27 | P27CF | Gangolli | Rural | Kundapura | Parents | - | - | 35-40 | Female | 7 | 01:21:51 |
| 28 | P28CF | Gujjadi | Rural | Byndoor | Parents | - | - | 35-45 | Female | 10 | 01:02:16 |
| 29 | P42CF | Hosodu | Rural | Byndoor | Parents | - | - | 40-45 | Female | 7 | 01:12:07 |
| 30 | P43CM | Sanoor | Rural | Karkala | Parents | - | - | 45-50 | Male | 8 | 01:06:49 |
| 31 | P44CF | Karje | Rural | Brahmavara | Parents | - | - | 30-40 | Female | 7 | 01:26:07 |
| 32 | P47CM | Kokkarne | Rural | Brahmavara | Parents | - | - | 40-50 | Male | 7 | 01:25:29 |
| 33 | T18PM | Hebri | Urban | Karkala | **Teachers** | Private | - | 35-40 | Male | 8 | 01:17:52 |
| 34 | T20AMF | Byndoor | Urban | Byndoor | Teachers | Aided | - | 23-55 | Male Female | 8 | 01:22:20 |
| 35 | T23PMF | Brahmavara | Urban | Brahmavara | Teachers | Private | - | 29-53 | Male Female | 10 | 01:29:27 |
| 36 | T25PM | Uchila | Rural | Udupi | Teachers | Private | - | 24-39 | Male | 8 | 01:19:15 |
| 37 | T26GMF | Koteshwara | Rural | Kundapura | Teachers | Government | - | 41-56 | Male Female | 8 | 01:47:32 |
| 38 | T29PMF | Uppunda | Rural | Byndoor | Teachers | Private | - | 26-37 | Male Female | 8 | 01:27:21 |
| 39 | T39PMF | Karkala | Urban | Karkala | Teachers | Private | - | 27-54 | Male Female | 7 | 01:18:04 |
| 40 | T40GMF | Kundapura | Rural | Kundapura | Teachers | Government | - | 25-52 | Male Female | 10 | 01:14:04 |
| 41 | T45PMF | Kanarpady | Rural | Udupi | Teachers | Private | - | 24-50 | Male Female | 8 | 01:10:23 |
| 42 | T46PMF | Brahmavara | Urban | Brahmavara | Teachers | Private | - | 29-35 | Male Female | 8 | 01:12:19 |
| 43 | PC30M | Udupi Block | **-** | Udupi | **Police** | - | - | 40-45 | Male | 8 | 01:21:32 |
| 44 | PC31MF | Byndoor Block | **-** | Byndoor | Police | - | - | 35-45 | Male Female | 9 | 01:09:55 |
| 45 | PC32 MF | Kundapur Block | **-** | Kundapura | Police | - | - | 30-45 | Male Female | 9 | 01:19:17 |
| 46 | PC33 MF | Karkala Block | **-** | Karkala | Police | - | - | 30-40 | Male Female | 8 | 01:13:49 |
| 47 | PC34 MF | Brahmavara Block | **-** | Brahmavara | Police | - | - | 40-50 | Male Female | 8 | 01:19:13 |

| **Sl. No** | **IDI No.** | **Location** | **Type of Area** | **Educational Block Name** | **Type of IDI** | **Age** | **Gender** | **Duration of IDI (In Hrs)** |
| --- | --- | --- | --- | --- | --- | --- | --- | --- |
| 1 | 1 | Kadubettu | Urban | Udupi | Vendor | 62 years | Male | 00:27:01 |
| 2 | 2 | Kukkehalli | Rural | Karkala | Vendor | 55 years | Male | 00:30:59 |
| 3 | 3 | Tekkatte | Rural | Kundapura | Vendor | 35 years | Male | 00:29:36 |
| 4 | 4 | Byndoor | Rural | Byndoor | Vendor | 31 years | Male | 00:19:18 |
| 5 | 5 | Kanyana | Rural | Kundapura | Vendor | 42 years | Male | 00:38:54 |
| 6 | 6 | Adi Udupi | Urban | Udupi | Vendor | 43 years | Male | 01:01:08 |
| 7 | 9 | Karkala | Urban | Karkala | Vendor | 51 years | Male | 01:06:31 |
| 8 | 10 | Brahmavara | Rural | Brahmavara | Vendor | 34 years | Male | 00:34:15 |
| 9 | 11 | Byndoor | Rural | Byndoor | Vendor | 42 years | Male | 00:38:45 |
| 10 | 12 | Varamballi | Urban | Brahmavara | Vendor | 62 years | Male | 00:46:01 |
