# Supplementary material for: Current scenario, challenges and way forward for augmenting tobacco control policies and programs in India: a community-based qualitative study
Source: Glob Health Action. 2025 May 15;18(1):2491195. doi: 10.1080/16549716.2025.2491195 (PMC12082722; doi:10.1080/16549716.2025.2491195)
Supplement: Supplimentary File 1_.docx [file ZGHA_A_2491195_SM8622.docx]

# Adolescents:

**A. GENERAL QUESTIONS ON TOBACCO USE**

1. In general, what do you think about the use of tobacco?

- Do you think it is a problem in the society and to what extent?
- Do you think it is a problem in your surroundings?

1. What do you know about the different types of tobacco products being used in India?

*Probes: smoking and smokeless tobacco, new emerging tobacco products (e-cigarettes, Shisha etc.)*

1. What do you think are the most common reasons for starting tobacco use?

*Probes: Availability, accessibility, affordability etc.*

**B. TOBACCO USE AMONG YOUNG PEOPLE**

1. In general, what do you think about tobacco use among young people?

- Do you think it is a problem and to what extent?
- Out of 10 people of your age, how many do you think use tobacco?

*Probe*: Tap by gender (boys and girls); by school status (school going and out-of-school/drop-out); by SES.

- Any ideas on when they tend to start? (age of initiation)
- Why do they tend to smoke or use tobacco? (reasons for initiation)

*Probes:* Experimentation, fashion, availability, accessibility, affordability, peer pressure, stress, family reasons, role models etc.

- Where do they tend to smoke or use tobacco?

Probes: around school/home or any other place

- What type of tobacco they use the most?

*Probes:* Smoked forms, Smokeless forms, e-cigarettes (Tap into gender differences and SES differences)

- How open are people of your age admit that they use tobacco or have tried tobacco before?

What do you think about the role of media on tobacco use among people of your age?

*Probes: internet, movies, newspapers, magazines, other social media platform.*

What generally do you watch on T.V/Radio/Internet?

*Probes: movies, sports, music or cartoon etc.*

*Frequency of watching in a day?*

- Have you ever noticed any anti-tobacco messages while watching TV, cinema or in radio? How impactful these messages would be for young people?
- Have you noticed any actor/actress smoking or using smokeless tobacco in TV, movies or music video? What role these celebrities can play?

1. Would any of you like to share any examples where you have seen or experienced situations of being pressured to try or start smoking or using tobacco?
   - What would you do if one of your friends offers you tobacco?
   - If your close friend started using tobacco, how would you feel about that? What would you say to your friend?
   - How confident would you be in telling your friend not to start using tobacco? What would you say?

**C. CONSEQUENCES OF TOBACCO USE**

1. What do you know about the harms of using tobacco?

*Probes: health (short term, long term, physical, mental, behavioral), financial, social, environmental etc.*

Both smoking, smokeless tobacco use, new emerging products like e-cigarette, shisha etc.

1. From where have you gained the information about the harms of using tobacco?

*Probes*: media (Print, electronic-TV etc.), social media (Internet), school, any family member, friend etc.

**D. PREVENTION OF TOBACCO USE AND QUITTING TOBACCO**

1. In your opinion, who can motivate and help young people:

- To keep away/not to try tobacco? Can you think of how this could be done?
- To stop using tobacco? Can you think of how this could be done?

*Probes:* Film stars, sports stars, Youth role models, teachers, adult role models in community, religious leaders, schools, family members, role of government

1. How important do you think it is for people to stop smoking or using tobacco? What do you think are the benefits of quitting?
2. How confident would you be in telling your friend to quit tobacco (if you know that your friend is using tobacco)? What would you say?
3. Is it possible for you to convince your parents or any family member to quit tobacco? What would you say?
4. In your opinion, what is the best method of delivering information or messages to the people of your age about the harmful use of tobacco? What should be the content of these messages? Probes: Posters, health talks, competition

Where do you think young people should be able to go to get help to quit tobacco?

*Probes:* Quit line, school counsellor, counsellor at Adolescent Health Clinic, discuss with parents or family members etc.

**E. AWARENESS REGARDING THE EXISTING TOBACCO CONTROL LAWS/POLICIES**

1. What do you know about the?
2. Smoking in public places
3. Sale of tobacco products to minors? Are you aware of the age?
4. Sale of tobacco products outside the boundary wall of the school premises
5. Tobacco advertisements (T.V, print media etc.)
6. Tobacco imagery in media/smoke-free movies
7. Health spots and disclaimers during movies
8. Pictorial health warnings
9. Gutkha and other SLT ban in your state
10. Sale of tobacco products only through licensed shops
11. Ban on sale of loose cigarettes
12. Ban on ENDS

- What do you think about these laws and policies? In your opinion, how effective or impactful these policies are in preventing tobacco uptake?
- In your opinion, what role could the government have in tobacco control?

**F. TOBACCO CONTROL EFFORTS/ACTIVITIES IN SCHOOLS**

1. What efforts your school has made with regard to tobacco education? (if anything)

- What was covered? How was it done?

*Probes: Posters, films, talks, outreach programme, National Tobacco Control Programme etc.*

- What do you think about these initiatives? Did it change your views on tobacco? If so how (e.g. further reinforce intention not to smoke). Could they be improved?
- How important do you think it is for schools to address this issue?
- What would you think about involving your parents or families in such kind of programmes?

*Probes:* Take home booklet with activities to complete with your family members/siblings; campaigns, pamphlets, postcards etc.

**G. TOBACCO CONTROL EFFORTS/ACTIVITIES IN YOUR COMMUNITY**

1. Have you come across anything else e.g. within your community around tobacco control or prevention etc.? What do you think about these initiatives? Could they be improved?

*Probes: Posters, films, talks, outreach programme etc.*

1. Before we wrap up, do you have any final thoughts that you would like to share?

# Teachers:

**A. GENERAL QUESTIONS ON TOBACCO USE**

1. In general, what do you think about the use of tobacco?

*Probes: Do you think it is a problem among people and to what extent?*

*Among adults, among adolescents (girls, boys, early adolescent, late adolescents)*

1. What do you know about the different types of tobacco products being used in India?

*Probes: smoking and smokeless tobacco, new emerging tobacco products (e-cigarettes, Shisha etc.)*

1. In your opinion, what are the most common reasons that people start using tobacco?

*Probes: Availability, accessibility, affordability etc.*

**B. TOBACCO USE AMONG YOUTH**

1. In general, what do you think about tobacco use among youth?

- Do you think it is a problem among youth and to what extent?
- Any idea, how many tobacco users are there in your school?

*Probe: Students (boys and girls); by age (10-14 years and 15-19 years)*

- Any ideas on when they tend to start? (age of initiation)
- Why do they tend to smoke or use tobacco? (reasons for initiation)

*Probes:* Experimentation, fashion, availability, accessibility, affordability, peer pressure, stress, family reasons, role models etc.

- Where do they tend to smoke or use tobacco?

*Probes: around school/home or any other place*

- What type of tobacco they use the most?

*Probes: Smoked forms, Smokeless forms, ENDS*

*Tap into gender differences and SES differences*

- What do you think about the role of media on tobacco use among youth/adolescent?

*Probes: internet, movies, newspapers, magazines, other social media platform etc.*

**C. CONSEQUENCES OF TOBACCO USE**

1. As per your knowledge, what are the consequences or harms of using tobacco?

*Probes: Health (physical, mental, behavioral etc.), social, financial and any other.*

*Smoking, smokeless tobacco use, new emerging products (electronic cigarettes, Shisha etc.)*

**D. PREVENTION OF TOBACCO USE AND QUITTING TOBACCO**

1. In your opinion, what would be the most effective way(s) of informing young people about the problems and consequences of tobacco use?
2. What steps should be taken to prevent and reduce youth tobacco use?
   1. Can you think of how this could be done?
   2. Who can motivate and help young people to keep away/not try tobacco?
   3. Who can motivate and help young people to quit tobacco?

*Probes: Film stars, sports stars, Youth role models, teachers, adult role models in community, religious leaders, schools, family members, role of government, role of media, role of schools*

1. How important do you think it is for people to stop smoking or using tobacco?
2. What do you think are the benefits of quitting tobacco?
3. How confident would you be in telling your students to quit tobacco (if you know that he/she is using tobacco)? What would you say?

*Probes: Describe your personal experience with students*

Where do you think young people should be able to go to get help to quit tobacco?

Probes: Quit line, school counsellor, Counsellor at Adolescent Health Clinic, discuss with parents, teachers or family members etc.

1. In your opinion, what action/s can be taken at college level for prevention and control of tobacco use?

**E. TOBACCO CONTROL ACTIVITIES/EFFORTS IN SCHOOLS**

1. What efforts your school has made with regard to tobacco control? Please share (if any)
   - Have you ever discussed about tobacco issue with your students?
   - Any classroom discussion/activity or school level awareness activity?

*Probes: Posters, films, talks, outreach programme, National Tobacco Control Programme etc.*

- - Have you ever developed these activities yourself?
  - What sort of activities? Conducted by whom?
  - If so, who is responsible for planning such kind of activities in your school?
  - At what time point were these delivered?

Probe: once or more than once e.g. each academic year

- - How were these activities received by students?
  - Do you think there is a need to address such matters at school level and if so how and why do you think it is a need?
  - What role you as a teacher can play in creating awareness about tobacco?
  - How aware and comfortable are you talking to the students about tobacco?
  - What do you think about the need of trainings teachers to effectively delivers tobacco control messages to the students?
  - Is there a display of boards with the messages in your school?

*“Tobacco Free School” or ‘Tobacco Free Institution”*

*“No smoking area - smoking here is an offence”*

*“Sale of tobacco products within 100 yards of school is strictly prohibited) “*

- - Are you aware about any “Tobacco Control Committee” in your school? What is the role of this committee? How often does the meet? Key activities undertaken by this committee in your school?
  - What would you think about involving parents and families in such kind of programmes or initiatives?

*Probes: Take home booklets with activities to complete with parents/siblings/family members, campaign, pamphlets, postcards etc.*

**F. AWARENESS REGARDING THE EXISTING TOBACCO CONTROL LAWS/POLICIES**

1. What do you know about?
2. Smoking in public places
3. Sale of tobacco products to minors?
4. Sale of tobacco products outside the boundary wall of the school premises
5. Tobacco advertisements (T.V, print media etc.)
6. Tobacco imagery in media/smoke-free movies
7. Health spots and disclaimers during movies
8. Pictorial health warnings
9. Gutkha and other SLT ban in your state
10. Sale of tobacco products only through licensed shops
11. Ban on sale of loose cigarettes
12. ENDS ban in your state

- What do you think about these policies? How effective or impactful these policies or laws in preventing uptake?
- In your opinion, what role government can play for tobacco control?

1. Before we wrap up, do you have any final thoughts that you would like to share?

# Parents:

**A. GENERAL QUESTIONS ON TOBACCO USE**

1. In general, what do you think about the use of tobacco?

*Probes*: Do you think it is a problem among people and to what extent?

*Among adults, among adolescents (girls, boys, early adolescent, late adolescents)*

1. What do you know about the different types of tobacco products being used in India?

*Probes*: smoking, smokeless tobacco, new emerging products (electronic cigarettes, shisha etc.)

1. As per your knowledge, how many people in your area/community consume tobacco?

*Probe*: Adults (males and females); SES difference

1. In your opinion, what are the most common reasons to initiate or start tobacco use

among people?

*Probes:* Availability, accessibility, affordability, stress, family reasons, curb hunger etc.

**B. TOBACCO USE AMONG YOUTH**

1. In general, what do you think about tobacco use among youth?

- Do you think it is a problem among youth and to what extent?
- How many youths in your area where you live, do you think use tobacco?

*Probes*: Tap by gender (boys and girls); by age (10-14 years and 15-19 years); by school status (school going and out-of-school/drop-out); by SES.

- Any ideas on when they tend to start? (age of initiation)
- Why do they tend to smoke or use tobacco? (reasons for initiation)

*Probes:* Experimentation, fashion, availability, accessibility, affordability, peer pressure, stress, family reasons, role models etc.

- Where do they tend to smoke or use tobacco?

Probes: around school/home or any other place

- What type of tobacco they use the most?

*Probes:* Smoked forms or Smokeless forms

What do you think about the role of media on tobacco use among youth/adolescent?

Probes: Internet, movies, newspaper, magazines, social media etc.

**C. CONSEQUENCES OF TOBACCO USE**

1. What do you know about the consequences or harms of using tobacco?

*Probes*: Health (short and long term, physical, mental, behavioral), economic, financial, social and any other.

Smoking and smokeless tobacco use, new emerging products (electronic cigarettes, Shisha etc.)

1. In your opinion, what would be the most effective way(s) of informing young people about the consequences of using tobacco?

**D. PREVENTION OF TOBACCO USE AND QUITTING TOBACCO**

1. What steps should be taken to prevent and reduce youth tobacco use? Can you think of how this could be done?
2. Who can motivate and help young people to keep away/not try tobacco?
3. Who can motivate and help young people to quit tobacco?

*Probes:* Film stars, sports stars, Youth role models, teachers, adult role models in community, religious leaders, schools, family members, role of government, role of media.

1. How important do you think it is for people to stop smoking or using tobacco? What do you think are the benefits of quitting?
2. Where do you think people should be able to go to get help to quit tobacco?

What would be the most effective ways of motivating people to quit tobacco use?

*Probes:* Tobacco cessation services, Quit line, counsellor, any health professional, mCessation etc.

**E. AWARENESS REGARDING THE EXISTING TOBACCO CONTROL LAWS/POLICIES**

1. What do you know about?
2. Smoking in public places
3. Sale of tobacco products to minors? Are you aware of the age?
4. Sale of tobacco products outside the boundary wall of the school premises
5. Tobacco advertisements (T.V, print media etc.)
6. Tobacco imagery in media/smoke-free movies
7. Health spots or disclaimers during movies
8. Pictorial health warnings
9. Gutkha and other SLT ban in your state
10. Sale of tobacco products only through licensed shops
11. Ban on sale of loose cigarettes
12. ENDS ban
13. What do you think about these laws and policies? In your opinion, how effective or impactful these policies or laws are in preventing tobacco uptake?
14. In your opinion, what role government can play for tobacco control?

**F. TOBACCO CONTROL EFFORTS/ACTIVITIES IN SCHOOLS**

1. Are you aware about any kind of tobacco education being provided in your child’s school?

- Any idea what has been covered?

*Probes: Posters, films, talks, outreach programme, National Tobacco Control Programme etc.*

- What do you think about these initiatives/programmes? Did it change your child’s views on tobacco? If so how? Could they be improved?

Probe: reinforce intention not to smoke

- If no such programme, how important do you think it is for schools to address this issue?

Probes: If important, what are the various ways you think to address this issue?

- What would you think about involving parents and families in such kind of programmes/ initiative?

*Probes:* Take home booklet with activities to complete with your family members/siblings; pamphlets, postcards, whatsapp messages etc.

**G. TOBACCO FREE RULES/NORMS AT HOME**

1. Any rules/norms in your family or home regarding tobacco-free home? Please share
   - Have you ever discussed tobacco with your child, its harmful effects?
   - How to say No or refuse if someone offers?
   - How would you feel about your child using tobacco? Imagine if they did, how would go about helping them to stop?

*Probes: if they would seek any support e.g. from health professional, counsellor, quitline, tobacco cessation services etc.*

**H. TOBACCO CONTROL EFFORTS/ACTIVITIES IN COMMUNITY**

1. Have you come across any initiative around tobacco control or prevention in your community?

- What do you think about these initiatives?
- Who do you think they are aimed at?
- Could they be improved?

*Probes: Posters, films, talks, outreach programme etc.*

1. Before we wrap up, do you have any final thoughts that you would like to share?

# Police constables:

**Information to be collected through short questionnaire, prior to FGD**

- Age
- Education Qualification
- How long have you been in this role?
- What are your key job responsibilities?
- How many hours in a day you work?
- Your responsibilities related to tobacco control?
- Have you attended any training on tobacco control organized by your department or any other department? Why did you have to attend? What was it about? What was the duration of the training? Who organized the training? How do you think it was useful to you?

**A. GENERAL QUESTIONS ON TOBACCO USE**

1. In general, what do you think about the use of tobacco?

*Probe*: Do you think it is a problem among people and to what extent?

*Among adults, among adolescents (girls, boys, early adolescent, late adolescents)*

1. What do you know about the different types of tobacco products being used in India?

*Probe*: smoking and smokeless tobacco, ENDS

**B. TOBACCO USE AMONG YOUTH**

1. In general, what do you think about tobacco use among young people?

- Do you think it is a problem among young people and to what extent?

*Probe: How many young people do you think use tobacco?*

*Tap by gender (boys and girls); by school status (school going and out-of-school/drop-out); by SES.*

- Any ideas on when they tend to start? (age of initiation)
- Why do they tend to smoke or use tobacco? (reasons for initiation)

*Probes: Experimentation, fashion, availability, accessibility, affordability, peer pressure, stress, family reasons, role models etc.*

- Where do they tend to smoke or use tobacco?

Probes: around school/home or any other place

- What type of tobacco they use the most?

*Probes:* Smoked forms or Smokeless forms (Tap into gender differences and SES differences)

What do you think about the role of media on tobacco use among youth/adolescent?

*Probes: Internet, movies, newspaper, magazines, social media etc.*

**C. CONSEQUENCES OF TOBACCO USE**

1. What do you know about the harms or consequences of using tobacco?

*Probes: Health impacts (physical, mental and behavioral), economic, financial, social and any other (smoking, smokeless, ENDS, Shisha etc.)*

**D.PREVENTION OF TOBACCO USE AND QUITTING TOBACCO**

1. How important do you think it is for people to stop smoking or using tobacco?
2. What do you think are the benefits of quitting?

**E. EXISTING TOBACCO CONTROL EFFORTS IN STATE/DISTRICT AND KEY CHALLENGES AND OPPORTUNITIES**

1. What tobacco control measures have been undertaken by your state/district?

- What are they meant to address? How effective are these?
- In your opinion, how much priority is by the government to prevent tobacco uptake among adolescents in your state and district?

*Probe: If not, what needs to be done?*

- What do you know about the National Tobacco Control Programme (NTCP) and its activities?

*Probes: Training of key stakeholders; IEC activities; Monitoring of tobacco control laws; School Programme; Coordination with Panchayati Raj Institutions for village level activities; Tobacco cessation facilities at district level.*

- Is there any Task Force/ District Level Coordination Committee to monitor the implementation of tobacco control activities in your district?

1. Are you member of this Task force/ Committee?
2. Who is heading this Committee?
3. What is the structure and membership of this Task Force/Committee?
4. Can you please elaborate on the roles and responsibilities of this task Force/Committee?
5. How frequently the committee meets?
6. How effective is the committee?
7. Are you aware of any enforcement squads/ teams responsible to monitor violation of COTPA provisions?
8. Any data on challans/ raids conducted?
9. On an average, how many challans issued in a month by enforcement squads/teams?
10. Approximate challan amount collected in a month? Mostly for which offence?
11. For what purpose, these funds utilized?
12. Does the Committee responsible for maintaining record or preparing any report of these violations?
13. What is your experience in implementation of these tobacco control policies?

*Probes:*

1. Ban of smoking in public places (smoke-free; Section 4 of COTPA)
2. Sale of tobacco products to minors (under 18 years of age); minor access; Section 6a of COTPA
3. Sale of tobacco products outside the boundary wall of the school premises; Section 6b of COTPA
4. Ban on tobacco advertisements (T.V, print media etc.); TAPS; Section 5 of COTPA
5. Licensing
6. Sale of tobacco products only through licensed shops
7. Gutkha and other SLT ban in your state
8. Pictorial health warnings (Section 7 of COTPA
9. Ban on sale of loose cigarettes
10. Tobacco imagery in media/smoke-free movies
11. Health spots or disclaimers during movie
12. ENDS ban
13. What is your opinion on the level of enforcement of these policies in your state/district?
14. What are the current opportunities and enablers for successful implementation of tobacco control policies?
15. What are the current gaps/challenges/barriers for successful implementation of tobacco control policies? How can these challenges be overcome?
16. What are your views on better implementation of existing tobacco control policies in your district?
17. In your opinion, who should be responsible for addressing tobacco uptake prevention/use among adolescents?

- What steps should be taken to prevent and reduce youth tobacco use? How this could be done?
- Where do you think people should be able to go to get help to quit tobacco?
- Who can motivate and help young people to keep away/not try tobacco?

*Probes: Role of schools, families/parents, government/police department, media, celebrities etc.*

1. What would be the most effective ways of motivating people to quit tobacco use? Who can motivate and help young people to quit tobacco?
2. Before we wrap up, do you have any final thoughts that you would like to share?

# Tobacco Selling Vendors:

1. How long have you been working in retail? Do you have the license to open the shop? What all products do you sell?
2. How do you feel about selling tobacco?
   1. From how long you are selling tobacco products?
   2. Have you ever considered not selling tobacco?
   3. What proportion of your profit comes directly from tobacco?
3. In general, what do you think about the use of tobacco? Do you use any tobacco products yourself?

*Probe: Do you think it is a problem among young people and to what extent?*

1. What do you know about the harms or consequences of using tobacco? What are the impact on health?

*Probes*: *Health (Physical, mental, behavioral*), financial, social, other consequences

Smoking, smokeless tobacco, new emerging products (e-cigarettes, shisha etc.)

1. What do you think are the most common reasons for adolescents to initiate/start tobacco use?
2. In your opinion, who should be responsible for addressing tobacco uptake prevention/use among adolescents?

- What steps should be taken to prevent and reduce youth tobacco use? How this could be done?
- Who can motivate and help young people to keep away/not try tobacco?

*Probes*: Role of schools, families/parents, government/police department, media, celebrities etc.

- Where do you think people should be able to go to get help to quit tobacco?

1. What would be the most effective ways of motivating people to quit tobacco use? What do you think are the benefits of quitting? Who can motivate and help young people to quit tobacco?

*Probes:* Tobacco cessation services, Quit line, counsellor, health professional, mCessation etc.

1. Can you tell us about how the sale of tobacco products has changed in recent years? If gone up or gone down, what do you think are the reasons for this?

*Probes:* Prices, pictorial health warnings, quit line number on the packs, awareness

Whether patterns differ according to age group (adults/adolescents: early and late adolescents)

Whether patterns differ according to gender (males/females)

1. Are you aware of the Indian Tobacco Control Act, 2003? What are the various provisions?

Probes:

1. Ban of smoking in Public places
2. Sale to minors
3. No sale within 100 yards
4. Tobacco advertisement, Promotion and sponsorship; Point of sale advertisements
5. Pictorial health warnings
6. Presence of specified health warning on the advertisement board
7. Ban on sale of loose cigarettes
8. ENDS ban
9. What is the minimum age to whom the tobacco products can be sold as per the Indian law? What do you think about this? How do you go about enforcing this?
10. Do you have Board stating/displayed that “Sale of tobacco products to a person below 18 years of age is a punishable offence”? Have you encountered any problems enforcing this?
11. Are you aware of the minimum distance to open tobacco selling shop from any educational institution (schools/colleges)? and what do you think about this?
12. What do you know about the point of sale advertisement? What should be the specification of displaying advertisement boards (if any), at point of sale as per the Indian law? What do you think about this?

*Probes: size, non-backlit, no brand name, and no promotional message*
